# A Point Mutation in Translation Initiation Factor eIF2B Leads to Function- and Time-Specific Changes in Brain Gene Expression

**DOI:** 10.1371/journal.pone.0026992

**Published:** 2011-10-31

**Authors:** Liraz Marom, Igor Ulitsky, Yuval Cabilly, Ron Shamir, Orna Elroy-Stein

**Affiliations:** 1 Department of Cell Research and Immunology, George S. Wise Faculty of Life Science, Tel Aviv University, Tel Aviv, Israel; 2 Blavatnik School of Computer Science, Tel Aviv University, Tel Aviv, Israel; 3 Interdisciplinary School of Neuroscience, Tel Aviv University, Tel Aviv, Israel; Newcastle University, United Kingdom

## Abstract

**Background:**

Mutations in eukaryotic translation initiation factor 2B (eIF2B) cause Childhood Ataxia with CNS Hypomyelination (CACH), also known as Vanishing White Matter disease (VWM), which is associated with a clinical pathology of brain myelin loss upon physiological stress. eIF2B is the guanine nucleotide exchange factor (GEF) of eIF2, which delivers the initiator tRNA^Met^ to the ribosome. We recently reported that a R132H mutation in the catalytic subunit of this GEF, causing a 20% reduction in its activity, leads under normal conditions to delayed brain development in a mouse model for CACH/VWM. To further explore the effect of the mutation on global gene expression in the brain, we conducted a wide-scale transcriptome analysis of the first three critical postnatal weeks.

**Methodology/Principal Findings:**

Genome-wide mRNA expression of wild-type and mutant mice was profiled at postnatal (P) days 1, 18 and 21 to reflect the early proliferative stage prior to white matter establishment (P1) and the peak of oligodendrocye differentiation and myelin synthesis (P18 and P21). At each developmental stage, between 441 and 818 genes were differentially expressed in the mutant brain with minimal overlap, generating unique time point-specific gene expression signatures.

**Conclusions:**

The current study demonstrates that a point mutation in eIF2B, a key translation initiation factor, has a massive effect on global gene expression in the brain. The overall changes in expression patterns reflect multiple layers of indirect effects that accumulate as the brain develops and matures. The differentially expressed genes seem to reflect delayed waves of gene expression as well as an adaptation process to cope with hypersensitivity to cellular stress.

## Introduction

Childhood Ataxia with Central nervous system Hypomyelination (CACH), also known as Vanishing White Matter disease (VWM), is an autosomal recessive genetic leukodystrophy associated with mutations in any one of the five subunits of eukaryotic translation initiation factor 2B (eIF2B) [1], [2]. The classical form of CACH/VWM is manifested during early childhood as progressive motor and cognitive impairments that ultimately lead to death by adolescence. Onset of signs and symptoms usually follows exposure to various environmental stressors, such as febrile illness, minor head trauma and acute fright, which also lead to exacerbation of symptoms during the course of disease progression. The diagnosis of CACH/VWM is based on MRI scans showing decreased brain white matter signals. The disease predominantly affects oligodendrocytes and astrocytes, while neurons are relatively preserved [3]–[9]. An R136H mutation in the human *EIF2B5* gene, encoding the catalytic subunit of eIF2B, is known to cause the classical form of CACH/VWM when present in a homozygous state. We recently generated a mutant mouse model for CACH/VWM disease by introducing an R132H mutation into the mouse *EIf2b5* gene locus, which corresponds to the R136H mutation in the human gene. The mutant mice exhibit delayed development of brain white matter, higher proportion of small-caliber nerve fibers, abnormal abundance of oligodendrocytes and astrocytes, specifically in young animals, and abnormal levels of major myelin proteins. Moreover, the mutant mice failed to recover from cuprizone-induced demyelination, reflecting an increased sensitivity to brain insults and difficulty in repairing damaged myelin [10].

eIF2B is the guanine nucleotide exchange factor (GEF) of translation initiation factor eIF2, which in its GTP-bound form binds aminoacylated initiator methionyl-tRNA to form the eIF2-GTP-tRNA_i_^Met^ ternary complex. The formation of ternary complexes directly depends on eIF2B, which recycles the inactive GDP-eIF2 back to its active GTP-eIF2 form following release from the ribosome at each round of translation initiation [11], [12]. eIF2B serves as a central regulatory hub governing global protein synthesis rates by responding to forms of cellular stress including starvation, viral infection, heat shock, accumulation of unfolded proteins in the ER, changes in intracellular calcium levels and oxidative stress, which activate one of four kinases that phosphorylate the α-subunit of eIF2 [13]. Phosphorylated eIF2 is a strong competitive inhibitor of eIF2B; given that eIF2B is significantly less abundant than eIF2, low levels of phosphorylated eIF2 are sufficient to effectively inhibit eIF2B activity, resulting in a significant decrease in global translation [14], [15].

Our previous results indicating abnormal brain development of the *Eif2b5*-mutated mice urged us to further explore the molecular mechanism responsible for the delayed white matter formation during the first three critical postnatal weeks. For this purpose, we conducted a genome-wide transcriptome analysis at three early postnatal stages of wild-type and mutant mice homozygous for the *Eif2b5* R132H mutation. The data reveal a massive effect of the point mutation in *EIf2b5* on global gene expression in the brain and provide a plausible explanation of the severity of CACH/VWM disease, despite the “mere” 20% reduction in eIF2B enzymatic activity associated with this specific mutation [10]. The largely disjoint differential gene expression signatures at the different time points suggest that *EIf2b5* mutation may lead to delayed brain development [10] by delaying waves of gene expression. The overall changes in gene expression patterns in the mutant mice may reflect multiple layers of indirect effects that accumulate as the brain develops and matures. This is most probably the result of a slight decrease in the translation efficiency of mRNAs that encode key regulators, e.g., transcription factors, components of the RNA processing machinery, and RNA binding proteins that affect mRNA stability. The identity of these regulatory factors, and how their expression level affect the fine tuning of brain development, is yet to be identified.

## Results

### A point mutation in *Eif2b5* affects the expression of distinct sets of genes at different developmental stages

**Figure 1:**
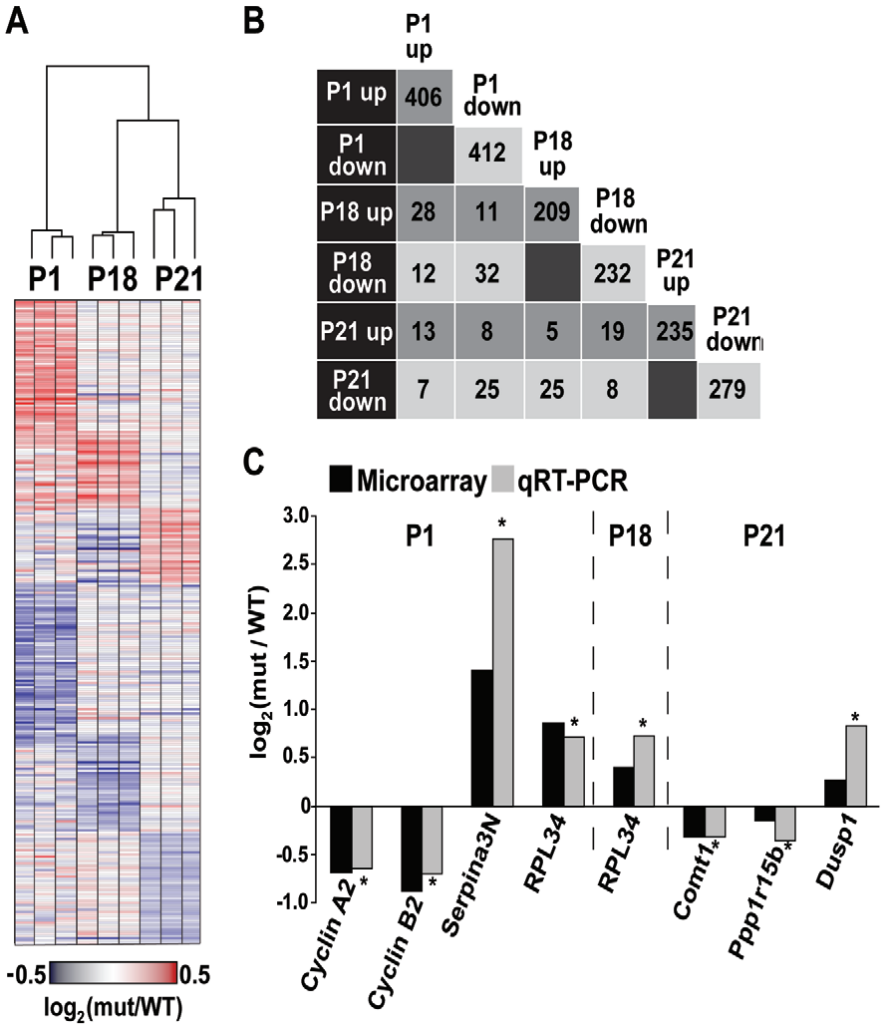
Differential gene expression at three postnatal time points. RNA extracted from the entire brain (cerebellum excluded) of wild-type and mutated mice at P1, P18 or P21 was analyzed for whole genome mRNA expression level by Affymetrix microarrays. File S1 lists all the differentially expressed genes. (A) Heatmap of relative gene expression in mutant mice when compared to wild type. For each mutant mouse, the expression level relative to the average of the three WT mice is shown. For visualization purposes, only genes exhibiting fold-change >1.35 (p<0.05) are shown. Red indicates higher expression in the mutant and blue indicates higher expression in the wild type. The dendogram shows a hierarchical clustering of the expression levels of all the genes using average linkage. (B) The number of genes that are differentially expressed at two different time points (fold-change >1.2; p<0.05). ‘up’ and ‘down’ refer to elevated or lower expression level, respectively, in the mutant relative to the wild-type mice at each time point. (C) Validation of selected differentially expressed genes. For validation, RNA was extracted from additional brains of wild-type and mutant mice at each developmental time point. Ratio of mRNA levels [log_2_(mutant/wild-type)] of the specific indicated genes determined by Affymetrix microarrays is shown (black) alongside with its level as detected by qRT-PCR and calculated relative to GAPDH control (gray). The qRT-PCR data represents the average of 3 biological samples (p<0.05).

In a previous study, we reported that the R132H mutation in the catalytic subunit of translation initiation factor 2B (eIF2B5) leads to delayed brain development in a mouse model for CACH/VWM disease [10]. To identify mutation-induced changes in overall gene expression in the mouse brain, we performed whole genome microarray analyses at different time points during the first three weeks of postnatal mouse brain development. Postnatal days 1, 18 and 21 were chosen to reflect early development prior to white matter establishment (P1) and the peak of oligodendrocye differentiation and myelin synthesis (P18 and P21) [16], [17]. For each time point, total RNA was isolated from the entire brain excluding the cerebellum of 3 wild-type and 3 mutant mice, followed by genome-wide measurement of mRNA expression by Affymetrix microarray (Mouse Genome 1.0 ST). At each time point, between 441 and 818 genes were differentially expressed in the *eIf2b5* R132H mutant mice (fold-change >1.2; t-test p<0.05, Figure 1A and File S1). There was surprisingly little overlap between the sets of genes dysregulated at different time points (Figure 1B). The differential expression of a total of 7 representative genes was validated by qRT-PCR (Figure 1C). The unique time-point-specific differential gene expression signature suggests that the altered global protein synthesis in mutant mice elicits a unique response depending on the developmental stage of the brain.

### Expression of cell-cycle related genes is low in mutant mice during early postnatal brain development

**Figure 2:**
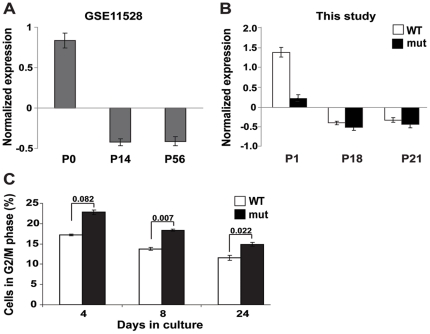
Expression of cell cycle-related genes. Average expression patterns of the 44 cell cycle-related genes with lower expression level in mutant mice at P1 (see Materials and Methods). (A) Expression during normal mouse brain development according to the measurements in [19].(B) Expression according to the current study. The expression pattern of each gene was normalized to mean = 0 and standard deviation = 1. The average expression of the genes in all replicates is shown for each time point. Error bars represent one standard error. (C) Primary astrocytes were isolated from wild-type (WT) and mutant (mut) mice brains at P1. Following 4, 8 and 24 days in culture, the cells were subjected to flow cytometry analysis using propidium iodide for DNA staining. The precentage of cells in G2/M phase is shown for wild-type (WT, white) and mutant (mut, black) primary astrocytes, representing the average of 3 independent experiments. t-test p values are indicated.

Each set of differentially-expressed genes was analyzed for enrichment of gene sets known to share a common function or gene sets previously reported to share common expression patterns during mouse development (see Materials and Methods; Table 1 and File S2 for complete enrichment results). Interestingly, the gene set differentially expressed at P1 was enriched with genes related to cell-cycle progression, whereas the gene set differentially expressed at P21 was enriched with oligodendrocyte-specific genes. Of the 44 cell-cycle associated genes the expression of which was low in the mutant brain at P1, 11 were related to mitosis (Table 1). During early postnatal stages, brain cells undergo multiple divisions [18]. Thus, lower expression level of mitotic genes may adversely affect cell proliferation during this critical developmental stage. This is consistent with the recently-reported delayed brain development of *Eif2b5*-mice [10]. Interestingly, during normal mice brain development (in *Mus musculus* WSB/EiJ strain), all 44 cell-cycle associated genes are highly expressed immediately after birth (P0) and down-regulated thereafter (Figure 2A, data extracted from GEO series GSE11528 [19], t-test p = 4.4·10^−26^). A similar trend was observed in the current study using wild-type (C57BL strain) mice, in which these specific genes were highly expressed at P1 and then down-regulated at P18 and P21 (Figure 2B, white bars [19], t-test p = 2.2·10^−27^). However, in mutant mice, the expression level of each of these genes was significantly lower at P1 (p<0.05), indicating that *Eif2b5* mutation either suppresses the up-regulation of cell-cycle associated genes immediately after birth or induces their premature down-regulation at P1 instead of at a later time point (Figure 2B, black bars). The lower level of two mRNAs, cyclin A2 and cyclin B1, was further validated by qRT-PCR (Figure 1C, grey bars). Since both cyclin A2 and cyclin B1 are required for progression through mitosis, their decreased expression level is expected to prolong mitosis [20]. To assess the progression of *Eif2b5*-mutated cells through the cell cycle, primary astrocytes were isolated from the brains of wild type and mutant newborn (P1) mice and subjected to flow cytometry analysis following propidium iodide staining of their DNA. It is expected that for a non-immortalized culture, the percentage of dividing cells will decline while the length of their G1 phase will increase, with time. Therefore, we expected to see more cells in G1/G0 and fewer cells in G2/M, as the cell culture gets older. This is indeed what was observed, for both WT and mutated primary astrocytes. However, at all time points tested, the FACS analysis demonstrated that significantly higher proportion of *Eif2b5*-mutated primary astrocytes were in G2/M phase (on the expense of the G1 phase, data not shown) compared to the WT cells. This indicates that the G2/M phase is significantly prolonged due to the mutation in *Eif2b5* (Figure 2C).

**Table 1:** Enrichments of co-annotated genes among the differentially expressed genes.

	Total no.	Enrichment category	No. genes	p-value
P1	Up regulated genes: 406	Localization: Dendrite	17	2.94·10^−5^
		Neuronal genes (GSE9566)	35	5.63·10^−6^
		Blood circulation	6	1.00·10^−3^
	Down regulated genes: 412	Localization: chromosome	28	2.40·10^−8^
		Cell cycle	44	3.72·10^−11^
		M phase	27	3.13·10^−10^
		Glycolysis/Gluconeogenesis	5	6.00·10^−3^
P18	Up regulated genes: 209	P16 and P26 neuronal genes (GSE9566)	14	8.90·10^−9^
		Retinal binding	3	2.04·10^−6^
		Positive regulation of T cell differentiation	3	0.007
	Down regulated genes: 232	Localization: Intracellular organelle	126	1.11·10^−6^
		Glial cell migration	2	0.004
P21	Up regulated genes: 235	Neuronal genes (GSE9566)	26	1.02·10^−5^
		Astrocytes genes (GSE9566)	27	3.84·10^−7^
		Innate immune response	18	1.59·10^−4^
	Down regulated genes: 279	Localization: extracellular region	34	1.18·10^−7^
		Mixed oligodendroglia genes (GSE13379)	55	2.97·10^−21^
		Oligodendrocytes genes (GSE9566)	52	1.04·10^−31^
		Astrocytes genes (GSE9566)	29	1.18·10^−6^
		Astrocytes genes (GSE13379)	19	1.19·10^−7^
		Iron ion binding	17	1.87·10^−6^
		Oxidoreductase activity	11	1.32·10^−6^
		Sterol metabolism	14	1.96·10^−11^
		Axon ensheathment	7	5.91·10^−6^

Differentially expressed gene sets were compared to Gene Ontology biological process annotations [50] and KEGG pathway annotations [51] using the hypergeometric distribution and corrected for multiple testing using the Benjamini and Hochberg FDR method [52]. They were also compared to two brain cell type-specific gene expression datasets [21], [53] which refer to GEO accessions GSE9566 and GSE13379. For each dataset, we first clustered the gene expression patterns using CLICK [22], manually annotated each cluster based on each expression pattern; and then tested the significance of the overlap between each set of differentially expressed genes in our data and each co-expression cluster using the hypergeometric test. Full gene lists of enrichment results are presented in File S2.

### Oligodendrocyte-specific genes are repressed in mutant mice during the peak of myelin formation

**Figure 3:**
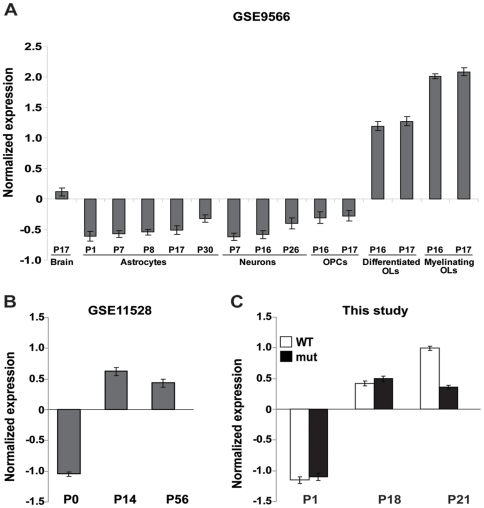
Expression of oligodendtocyte-related genes. Differential expression of the 52 genes with lower expression level in mutant mice at P21 that are also co-expressed in oligodendrocytes according to GEO accession GSE9566 [21] (see Materials and Methods). The expression pattern of each gene was normalized to mean = 0 and standard deviation = 1. (A) The average expression pattern of these genes in various brain cell types at various developmental time points as measured in [21]. (B) The average expression pattern of these genes during normal mouse brain development as measured in [19]. (C) The average expression pattern of these genes according to the current study. Error bars show one standard error.

Comparison of our data with expression dataset from neuronal cell types (GSE9566, [21], clustered using CLICK [22]) revealed a highly-significant overlap between the genes repressed in *Eif2b5*-mutated mice at P21 and genes that are highly expressed in oligodendrocytes (see Materials and Methods and Figure 3A, p = 1.06·10^−31^). The latter set of genes was also enriched in genes with decreased expression level at P18, but to a lesser extent (p = 0.009). Such specific enrichment suggests that the mutation in *Eif2b5* negatively affects specific oligodendrocyte functions at postnatal days 18 and 21, considered the peak period of myelin formation [16], [17]. We focused on 52 genes of the oligodendrocyte-specific cluster with lower expression level at P21 in the mutants. During normal brain development of mice (in *Mus musculus* WSB/EiJ strain), the expression level of these genes is low immediately after birth (P0), increases by P14 and remains high at P56 (data extracted from GEO series GSE11528 [19]; see Figure 3B, t-test P = 7.5·10^−56^ for the difference between P0 and P14). A similar trend was observed with our wild-type mice, which exhibited a relatively low expression level of these genes at P1 followed by up-regulation by P18 and P21 (Figure 3C, white bars, t-test p = 5.6·10^−67^ for the difference between day 1 and day 18). However, in contrast to wilt-type mice, the expression level of each of these genes in *Eif2b5*-mutated mice was significantly lower at P21 (p<0.05), indicating abnormal down-regulation at this time-point (Figure 3C, black bars). This pattern is consistent with the delayed brain development associated with the point mutation in *Eif2b5*, as reported earlier [10].

### Temporal dynamics of differential expression in the mutant mouse brain

**Figure 4:**
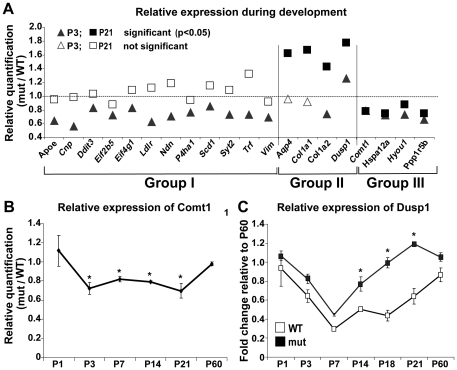
qRT-PCR of selected genes during development. RNA was isolated from the entire brain excluding the cerebellum of wild-type (WT) and mutant (mut) mice at different postnatal stages as indicated, and tested for specific mRNAs level by qRT-PCR using specific primers. Each mRNA expression level is the average of 3 biological samples calculated relative to GAPDH control. (A) P3, triangles; P21, squares. p<0.05, black; p>0.05, white. See Table 2 for exact p values of all samples. (B) Relative quantity of Comt1 mRNA level in mut relative to WT mice at P1, P3, P7, P14, P21 and P60. * indicates p<0.05. (C) Fold change of Dusp1/MPK1 expression level in WT (white) and mut (black) mice at P1, P3, P7, P14, P21 relative to P60. * indicates p<0.05.

Next, we selected 39 genes for further validation by qRT-PCR, focusing on postnatal days P3 and P21 (Table 2). Of the 39 genes, the change in expression of 20 genes was validated (Table 2, bold) and these were divided into three groups based on their expression pattern (Figure 4A). Group I consists of 12 genes that were down regulated in the mutant mice at P3 (Figure 4A, black triangles) but normally expressed at P21 (Figure 4A, white squares). This group includes two translation initiation factors (*Eif4g* and *Eif2b5*), a major myelin protein 2′,3′-Cyclic-nucleotide 3′-phosphodiesterase (Cnp), *Ddit3* and genes related to lipid metabolism and transport (*Apoe, Ldlr, Scd1*). Group II consists of 4 genes that were up-regulated in the mutant mice at P21 (Figure 4A, black squares); of these, 2 (*Aqp4* and *Col1a1*) were normally expressed at P3 (Figure 4A, white triangles) while *Col1a2* was down-regulated and *Dusp1* (also known as *Mkp1*) was up-regulated at P3 (Figure 4A, black triangles). Group III consists of *Comt1, Hspa12a, Hyou1 and Ppp1r15b* (also known as *Crep*), all of which were down-regulated in the mutant mice both at P3 and P21 (Figure 4A, black squares and triangles). In order to dissect differential expression on a finer temporal scale, we selected 2 genes for further analysis, namely Catechol-O-methyltransferase 1 (Comt1) and Dual specificity protein phosphatase 1 (*Dusp1/Mkp1*), which were either down-regulated (*Comt1*) or up-regulated (*Dusp1/Mkp1*) at both P3 and P21 (Figure 4A). We monitored Comt1 and Dusp1/MPK1 mRNA levels in the brain of wild-type and mutant mice by qRT-PCR at several postnatal timepoints from P1 to P60. Interestingly, whereas no difference in Comt1 mRNA levels was observed between wild-type and mutant mice at P1, Comt1 mRNA level in mutant mice was down-regulated soon after birth (at P3) and remained lower compared to wild-type mice for at least 3 weeks (P7, P14 and P21), followed by normal levels in adult mutant mice (P60) (Figure 4B). This underlines the transient abnormality of Comt1 gene expression during early postnatal brain development due to *Eif2b5* mutation. Analysis of Dusp1/Mkp1 mRNA levels also showed transient mutation-induced differences. Importantly, our analysis revealed that during normal brain development, Dusp1/Mkp1 expression is down-regulated in the first week after birth followed by gradual up-regulation in the second and third weeks until it returns to its initial high levels in the adult brain (Figure 4C, white squares). Dusp1/Mkp1 mRNA levels were similar in wild-type and *Eif2b5*-mutated mice during the first two postnatal weeks. However, while Dusp1/Mpk1 mRNA levels increased in a moderate fashion during the third postnatal week in the wild-type mice, its levels ascended more drastically in the mutant mice, building abnormal up-regulation specifically during the peak of white matter formation (Figure 4C, black squares).

**Table 2:** Temporal dynamics of differential gene expression.

	P3		P21		
Gene symbol	RQ	PV	RQ	PV	Gene Bank
Abca1	0.90	0.20	0.95	0.09	NM_013454.3
Adamts9	1.11	0.68	1.10	0.64	NM_175314.3
Adm	0.78	0.24	1.14	0.76	NM_009627
**Apoe**	**0.64**	**0.04**	0.95	0.69	NM_009696.3
**Aqp4**	0.96	0.66	**1.62**	**0.00**	NM_009700.2
Ccl21a	1.05	0.48	0.97	0.64	NM_011335.2, NM_011124.4, NM_023052.2
Cend1	0.94	0.12	1.00	0.99	NM_021316.3
Cldn11	1.03	0.54	1.04	0.79	NM_008770.2
**Cnp**	**0.56**	**0.02**	0.98	0.89	NM_009923.1
**Col1a1**	0.92	0.19	**1.67**	**0.05**	NM_007742.3
**Col1a2**	**0.74**	**0.01**	**1.43**	**0.05**	NM_007743.2
**Comt1**	**0.79**	**0.00**	**0.78**	**0.00**	NM_007744.3, NM_001111062.1 NM_001111063.1
**Ddit3**	**0.83**	**0.03**	1.03	0.63	NM_007837.3
Ddr2	0.97	0.82	1.14	0.31	NM_022563.2
**Dusp1**	**1.26**	**0.03**	**1.77**	**0.02**	NM_013642.3
Eif2ak2	1.21	0.14	0.97	0.80	NM_011163.4
**Eif2b5**	**0.72**	**0.02**	0.88	0.35	NM_172265
**Eif4g1**	**0.83**	**0.02**	1.09	0.28	NM_001005331.1 NM_145941.2
Ermn	0.95	0.67	1.23	0.15	NM_029972.3
Fasn	0.81	0.14	0.99	0.88	NM_007988.3
Gjc2	1.08	0.90	1.12	0.61	NM_080454.4 NM_175452.4
Gpm6a	0.88	0.22	0.86	0.16	NM_153581.3
Hhip	1.06	0.75	0.95	0.63	NM_020259.4
**Hspa12a**	**0.72**	**0.00**	**0.75**	**0.02**	NM_175199.3
**Hyou1**	**0.73**	**0.00**	**0.88**	**0.03**	NM_021395.3
Lcat	0.84	0.26	1.20	0.17	NM_008490.1
**Ldlr**	**0.63**	**0.01**	1.12	0.38	NM_010700
Mag	0.86	0.82	1.05	0.75	NM_010758.2
Mobp	1.09	0.92	1.09	0.36	NM_001039365.2 NM_008614.2
**Ndn**	**0.71**	**0.03**	1.18	0.17	NM_010882.3
Notch3	0.81	0.19	1.04	0.55	NM_008716
**P4ha1**	**0.76**	**0.01**	0.94	0.46	NM_011030.2
Plxnb3	0.78	0.12	0.96	0.75	NM_019587.2
**Ppp1r15b**	**0.66**	**0.00**	**0.75**	**0.05**	NM_133819.2
**Scd1**	**0.85**	**0.02**	1.15	0.43	NM_009127.4
Stab1	0.83	0.26	1.20	0.08	NM_138672.2
**Syt2**	**0.73**	**0.03**	1.09	0.60	NM_009307.3
**Trf**	**0.73**	**0.03**	1.32	0.16	NM_133977.2
**Vim**	**0.69**	**0.02**	0.92	0.49	NM_011701.3

RNA from cerebrum of wild-type and mutant mice at P3 and P21 was isolated and analyzed by qRT-PCR using specific primers. RQ = relative quantification of averaged 3 biological samples calculated relative to GAPDH control. Data is presented as expression level in mutant relative to wild-type mice. PV, p value; **Bold indicates a significant change (p<0.05).**

### Spatial dynamics of differential expression in mutant mice brain

**Figure 5:**
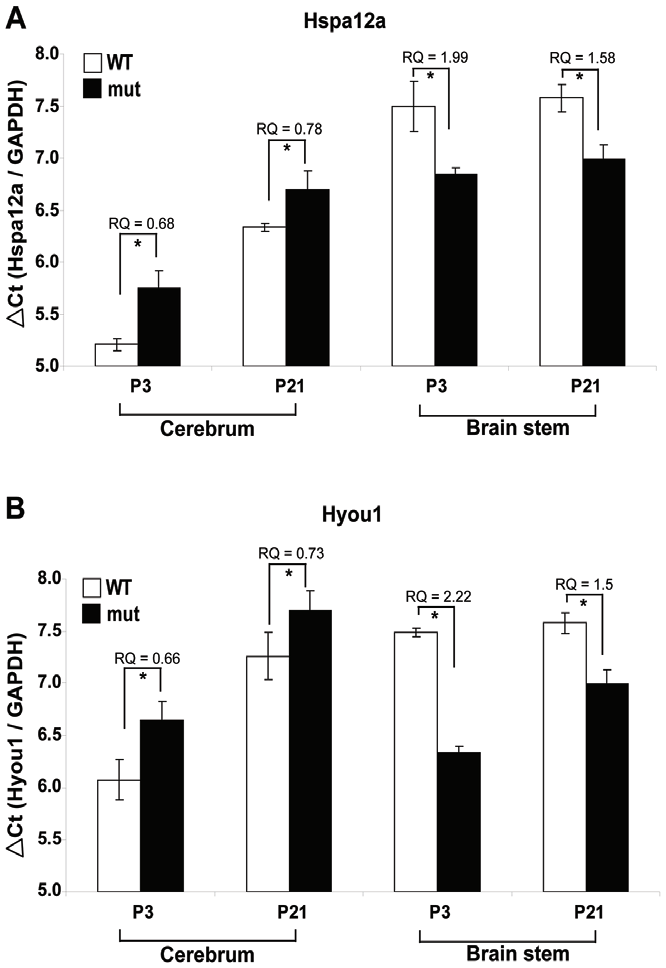
qRT-PCR of Hspa12a and Hyou1 in different brain regions. RNA was isolated from the cerebrum or brain stem of wild type (WT) and mutated (mut) mice at P3 and P21 followed by qRT-PCR analysis using primers specific for Hspa12a (A) and Hyou1 (B). Each mRNA expression level shown is the average of 3 biological samples calculated relative to GAPDH control. Data are presented as ΔCt (gene of interest/GAPDH). High ΔCt value indicates low mRNA level. The calculated relative quantity (RQ) in mut relative to WT is shown for each case; * indicates p<0.05.

To better understand the spatial distribution of abnormal gene expression, 12 genes were selected for further analysis of mRNA levels in the cerebrum and brain-stem of wild-type and *Eif2b5*-mutated mice at P21. The expression level of 2 of these genes (*Cend1, Gjc2*) was altered only in the brain stem but not the cerebrum, whereas the expression level of others (*Aqp4*, *Cola1*, *Cola2*, *Dusp1/Mkp1* and *Ppp1r15b/Crep*) was altered only in the cerebrum but not the brain stem. In contrast, the expression level of yet another group of genes (*Comt1*, *Hspa12a* and *Hyou1*) was altered in both brain regions (Table 3). Of these, *Comt1* was down-regulated in both regions while *Hspa12a* and *Hyou12* were up-regulated in the brain stem but down-regulated in the cerebrum at P21 (Table 3). To test if *Hspa12a* and *Hyou12* also share similar age-specific alterations, their mRNA levels were quantitated by qRT-PCR at P3 in both brain regions. This analysis revealed that at both time points during early postnatal development, the mutation in *Eif2b5* led to lower levels of Hspa12a and Hyou1 mRNAs in the cerebrum (as indicated by the higher ΔCt values, Figure 5) and higher levels of both mRNAs in the brain stem (as indicated by the lower ΔCt values, Figure 5).

**Table 3:** Spatial dynamics of differential gene expression at P21.

	Cerebrum		Brain stem		
Gene symbol	RQ	pv	RQ	pv	Gene Bank
**Cend1**	1.00	0.99	**0.82**	**0.03**	NM_021316.3
**Gjc2**	1.12	0.61	**1.66**	**0.02**	NM_080454.4
Ldlr	1.12	0.38	1.49	0.06	NM_175452.4NM_010700
Trf	1.32	0.16	1.52	0.06	NM_133977.2
**Aqp4**	**1.62**	**0.00**	1.47	0.07	NM_009700.2
**Col1a1**	**1.67**	**0.05**	0.99	0.92	NM_007742.3
**Col1a2**	**1.43**	**0.05**	1.14	0.25	NM_007743.2
**Dusp1**	**1.77**	**0.02**	0.96	0.70	NM_013642.3
**Ppp1r15b**	**0.75**	**0.05**	1.12	0.30	NM_133819.2
**Comt1**	**0.78**	**0.00**	**0.70**	**0.00**	NM_007744.3,NM_001111062.1NM_001111063.1
**Hspa12a**	**0.75**	**0.02**	**1.5**	**0.00**	NM_175199.3
**Hyou1**	**0.88**	**0.03**	**1.34**	**0.03**	NM_021395.3

RNA from cerebrum of wild-type and mutated mice at P21 was isolated and analyzed by qRT-PCR using specific primers. RQ = relative quantification of averaged 3 biological samples calculated relative to GAPDH control. Data is presented as expression level in mutant relative to wild-type mice. PV, p value; **Bold indicates a significant change (p<0.05).**

## Discussion

Brain development is the result of coordinated interactions between molecular networks and systematic cross-talk between certain cell types influencing the development of others via waves of transcription/translation of specific genes. During development, the brain undergoes typical stages involving proliferation, differentiation, myelination, synapse formation, and maturation of synaptic function [18], [23]–[25]. Research into the underlying molecular mechanisms led to a series of gene profiling studies revealing that during development, gene expression profiles undergo spatial and temporal changes that correlate with phenotypic functions [19], [26]–[29]. The study of Wen et al. [30] demonstrated that fundamental patterns of temporal fluctuations in gene expression during development can be determined even without the dissection of whole tissues into distinct anatomical sub-regions. Here we studied a mutant mouse homozygous for a point mutation in the catalytic subunit of the translation initiation factor eIF2B, which leads to a ∼20% decrease in its enzymatic activity. This mutant mouse is the first animal model for eIF2B-related leukodystrophy (also termed CACH/VWM disease). The disease in humans is manifested as impaired white matter functions, which deteriorate upon stress conditions to loss of axonal function, followed by death [5]–[7]. We recently demonstrated that the *Eif2b5*-mutated mice suffered from delayed brain development under normal conditions, a circumstance not associated before with human patients probably since it can only be detected in the pre-symptomatic stage [10]. The current study further demonstrates the profound effect of the seemingly-superficial impairment in eIF2B activity on the entire gene expression profile throughout early postnatal brain development. Both wild-type and mutant mice underwent similar changes in global gene expression, which underlie CNS transition from a primarily proliferative state during the first postnatal week to a highly differentiated state later on. However, the expression of cell-cycle associated genes at P1 and white matter-specific genes at P21 were significantly dysregulated in mutant compared to wild-type mice (Figures 2B, 3C). The unique time-point-specific differential gene expression signature (Figure 1, Table 1) together with the delayed brain development observed in *Eif2b5*-mutated mice [10], suggests that the mutation in *eIf2b5* is responsible for delayed waves of gene expression, resulting in prolonged development. The delay is not uniform across the transcriptome, but seems to affect specific subset of genes. This can be the result of a slight decrease in translation efficiency of mRNAs that encode key regulators e.g. specific transcription factors, RNA processing machinery components and/or RNA binding proteins that affect the steady-state level of a subset of gene products.

Obviously, the overall difference in gene expression patterns in the mutant mice may reflect multiple layers of indirect effects that accumulate as the brain develops and matures. The delayed wave of gliogenesis-specific gene expression beyond postnatal day 21 and the inexact steady-state levels of mRNAs within the wave is in agreement with the abnormal time-course MRI of the mutants. More specifically, while the fractional anisotropy parameter seemed to be pseudo-normalized at an older age, the abnormal diffusion coefficient parameter was indicative of tissue pathology [10]. Indeed, defective glial maturation was also found in the brains of CACH/VWM human patients [9], [31]. Interestingly, translation initiation factors (e.g. *eIf2b5, eIf4g1*) and multiple genes involved in lipid and cholesterol metabolism, as well as myelin formation (e.g. *Ldlr, Apoe, Scd1, Syt2, Cnp, Vim*), were down-regulated at P3 but not at P21 (Figure 4A, Table 2, File S2) emphasizing the outcome of early perturbation of gene expression on the whole-brain phenotype at a later stage despite pseudo-normalization of expression of certain genes at older ages. The transient differential expression of Comt1 serves as an additional example for the delayed white matter development. Comt1, an abundant protein in astrocytic processes surrounding synapses [32], is responsible for metabolizing catecholamine neurotransmitters following their uptake by glial cells (known as the less important uptake route, termed uptake_2_) [33]). The transient down-regulation of Comt1 in the mutant brain during early postnatal development (Figure 4B) is in agreement with the delayed differentiation of astrocytes observed in the mutants [10] and coincides with the defective generation of astrocytes in CACH/VWM human patients [9], [31].

Another set of differentially-expressed genes relates to stress response. Among these are *Hspa12a, Hyou1, Ddit3, Ppp1r15b/Crep, Dusp1/MKP1* and others (Figure 4A, Table 2, and File S2). It is of note that CACH/VWM disease is strongly associated with stress: (a) myelin loss and associated neurological symptoms of human patients deteriorate upon exposure to various stressors [4], [5], [6], [34]; (b) the unfolded protein response (UPR) pathway was found to be activated in the brains of CACH/VWM patients [35], [36]; (c) primary cultured fibroblasts isolated from CACH/VWM patients, as well as oligodendroglia derived-cell line expressing mutated eIF2B, are hypersensitive to induced ER stress [37], [38]; and (d) *Eif2b5*-mutated mice fail to recover from induced demyelination [10]. It is widely accepted that myelination is a stressful endogenous process, as differentiation of oligodendrocyte precursor cells to mature myelinating oligodendrocytes involves synthesis of large amounts of lipids and proteins, accompanied by UPR activation to reduce the high rate of protein misfolding. Unbalanced expression of UPR-related genes prevents efficient differentiation and may lead to apoptosis or mal-differentiation. Since mutations in eIF2B lead to hypersensitivity to stress, malfunctioning of the myelination process is not surprising and expected to reflect the net result of all adaptive mechanisms. In this respect, it is interesting to note that *Ppp1r15b/Crep* is down-regulated in the mutant mice at both P3 and P21 (Figure 4A, Table 2). Ppp1r15b/Crep promotes dephosphorylation of the translation initiation factor eIF2α to enable efficient protein synthesis [39]. Therefore, this down-regulation of Ppp1r15b/Crep in the brain may suggest a process of adaptation to prolong UPR length in order to allow sufficient differentiation rates. To support proper protein folding, increased chaperone activity is also required. Indeed, expression of Hspa12a and Hyou1 is up-regulated at both P3 and P21 in the mutant brain (Figure 4A). Both are members of the Hsp70 chaperone family, induced by thermal or oxidative stress to protect the cells from ER stress-induced apoptosis [40]–[42].

In rodents, the process of myelination in the CNS occurs essentially after birth, proceeds along a caudo-rostral axis, and is completed by the third postnatal week. During development, mature oligodendrocytes expressing markers such as myelin basic protein (MBP) accumulate first in the spinal cord and then in the diencephalon, the brainstem, the cerebellum, and finally the cerebral hemispheres [43]. Notably, Hspa12a and Hyou1 expression in the mutants is up-regulated in the brain stem but not in the cerebrum (Figure 5), which is most probably in synchrony with the level of gliogenesis and myelination in this white matter-rich sub-anatomical region. It is unclear why Hspa12a and Hyou1 expression is down-regulated in the cerebrum at both ages in the mutant compared to wild-type mice (Figure 5), but this may be of clinical significance as CACH/VWM symptoms are localized to the cerebrum, while the brainstem is preserved. Interestingly, Hspa12a also shows reduced expression in the prefrontal cortex of subjects with schizophrenia [44]. An additional notable differential expression in the mutant brain is that of Dusp1/Mkp1, which dephosphorylates threonine and tyrosine residues of MAP kinases and inhibits MAPK signaling [45]. It was recently shown that the intensity and duration of JNK signaling are essential determinants of cellular response to ER stress in neurons and neuronal stem cells. Whereas transient JNK activation is a protective event, prolonged JNK activation mediates pro-apoptotic signaling. Moreover, Li and co-workers demonstrated that JNK activation is tightly controlled in these cells through ER stress-mediated Dusp1/Mkp1 activation [46]. Here we found that Dusp1/Mkp1 expression level is gradually decreased in both wild-type and mutant brains from postnatal day 1 to day 7, followed by gradual increase as the brain develops towards adulthood. Strikingly however, the increased level during the second and third postnatal weeks is significantly higher in mutant mice (Figure 4C), suggesting that *Eif2b5*-mutated brains are forced to employ extra protective means during stressful periods such as times of differentiation, synaptogenesis and massive myelination.

The current study reveals the massive effect of a mild point mutation in eIF2B, a key translation initiation factor, on global gene expression in the brain. It provides a plausible explanation of the severity of CACH/VWM disease, despite the “mere” 20% reduction in eIF2B enzymatic activity associated with this mutation. Future experiments using system biology approaches will enable the discovery of the molecular circuits involved in this pathology and may provide the basis for rational drug design.

## Materials and Methods

### Mice maintenance and sample collection

For all testing, we used male wild-type and mutant siblings of heterozygote mice that were backcrossed to the C57BL strain. All experimental procedures involving mice were approved by the Tel Aviv University Animal Care Committee according to national guidelines (permit #L-09-35). Pups at different ages as indicated were collected from WT or *Eif2b5*-mutated mating cages, housed in an animal facility with a 14/10 h light/dark cycle in filtered-top cages supplemented with autoclaved wood chips in laminar flow hoods. Animals were fed autoclavable rodent pellet (Koffolk 19–510, Koffolk Ltd, Petach Tikva, Israel) and sterile water ad libitum throughout the experiments. Mice were decapitated followed by brain removal, separation to cerebrum, brain stem and cerebellum when needed and flash freezing in liquid nitrogen for further use. For all experiments, each sample represents the brain of a single mouse.

### Genotyping and sex determination

DNA was isolated from mice tails using GenElute Mammalian Genomic DNA Miniprep Kit (Sigma) and used for genotyping by PCR as described previously [10]. Newborn mice were tested for their sex by PCR using specific primers for SRY on the Y chromosome (SRY-Fwd 5′TCTTAAACTCTGAAGAAGAGAC3′ and SRY-rev 5′GTCTTGCCTGTATGTGATGG3′) and for NDS on the X chromosomes (NDS-Fwd 5′ GAGTGCCTCATCTATACTTACAG3′ and NDS-rev 5′TCTAGTTCATTGTTGAGTTGC3′) [47].

### RNA isolation

Total RNA from brain tissues was isolated using Trizol reagent (Invitrogen). RNA samples for microarray analysis were further cleaned using Phase Lock Gel (Eppendorf). RNA quality was assessed by its absorbance at 260 nm and 280 nm using a NanoDrop spectrophotometer and its integrity was assessed by agarose gel electrophoresis.

### Microarray and data analysis

The GeneChip Mouse Gene 1.0ST Array (Affymetrix, Santa Clara, CA), which interrogates 28,853 mouse genes across 770,317 distinct probes, was used for expression profiling. A single chip was used to profile the expression pattern of a single brain. A total of 18 chips were used (three WT and three mutant brains were used for each of three time points). Affymetrix GeneChip Whole Transcript Sense Target Labeling Assay Manual Version 4 was followed for generation and amplification of biotinylaed sense-strand DNA targets. Briefly, 300 ng total RNA underwent first-strand and second strand cDNA synthesis. Complementary RNA was generated and used to produce sense-strand cDNA, which was fragmented and end-labeled with biotin. Microarrays were hybridized, washed, stained, and scanned according to the protocol described in the WT sense target labeling assay manual from Affymetrix (version 4; FS450_0007). Microarray data were normalized using RMA [48]. Probes were mapped to Entrez Gene identifiers using BioMart [49]. Genes were defined as differentially expressed if the ratio between the average expression in three mutant and the three WT mice was at least 1.2 and *t*-test *p*-value was below 0.05. For visualization purposes (Figures 2 and 3), the expression pattern of each gene was normalized to mean = 0 and standard deviation = 1. All original data files from the microarray experiments have been deposited in the National Center for Biotechnology Information Gene Expression Omnibus (GEO, accession number GSE32201). File S1 contains all the differentially expressed genes (p<0.05). All data is MIAME compliant.

### Functional analysis of the differentially expressed genes

Differentially expressed gene sets were compared to Gene Ontology biological process annotations [50] and KEGG pathway annotations [51] using the hypergeometric distribution and corrected for multiple testing using the Benjamini and Hochberg FDR method [52]. In order to test if the differentially expressed genes were preferentially expressed in specific brain cell types, we also analyzed two brain cell type-specific gene expression datasets [21], [53] which refer to GEO accessions GSE9566 and GSE13379, respectively]. For each dataset, we first clustered the gene expression patterns using CLICK [22], manually annotated each cluster based on each expression pattern; and then tested the significance of the overlap between each set of differentially expressed genes in our data and each co-expression cluster using the hypergeometric test. Full gene lists of enrichment results (Table 1) are presented in File S2.

### cDNA preparation and quantitative real-time PCR

Following RNA isolation, RNA samples were further purified using DNase I (QIAGEN) to remove potential genomic DNA contamination, when TaqMan® PCR reactions were performed. For syber-green qRT-PCR, 1 µg of RNA was reversed-transcribed using Verso cDNA kit (Thermo scientific). qRT-PCR was carried out for 40 cycles in a Rotor-Gene 6000 (Corbett research) using primers for cyclin-B1 (cycB1-Fwd 5′CAGAGTTCTGAACTTCAGCCTG3′ and cycB1-rev 5′TTGTGAGGCCACAGTTCACCAT3′); cyclin-A2 (cycA2 Fwd 5′TGAGTTTGATAGATGCTGACCCG3′ and cycA2-rev 5′ ATCCAGTCTGTTGTGCCAATGAC3′); Serpina3n (ser3n-Fwd 5′GGGAAGTCTTCTCCACACAGG3′ and ser3n-rev 5′AATTTGACTCCAGTGGCAGCA3′); Comt1 (com-Fwd 5′GCTACTCAGCCGTGCGAATGG3′ and com-rev 5′ GCCACATTCCTCCAGGAGAAGTG3′); RPL34 (rpl34-Fwd 5′ AGAAGGTTGGGAAAGCACCT3′ and rpl34-rev 5′ATAAGGAAAGCCCGCTTGAT3′); and GAPDH which was used an internal control (GAPDH-Fwd 5′TGGCAAAGTGGAGATTGTTGCC3′ and GAPDH-rev 5′AAGATGGTGATGGGCTTCCCG3′). For TaqMan® PCR reactions, 1 µg of RNA was converted to cDNA using the High-Capacity cDNA Reverse Transcription Kit (Agentek, Applied Biosystems). qRT-PCR was carried out for 40 cycles in a StepOne Real-time PCR system (Applied Biosystems) using gene-specific TaqMan® Gene Expression Assay (Agentek, Applied Biosystems). We used TaqMan® for hspa12a (Mm00558341_m1), dusp1 (Mm00457274_g1), hyou1 (Mm00491279_m1), aqp4 (Mm00802131_m1), eIF4G1 (Mm00524099_m1) and GAPDH (Mm99999915_g1) as control. Additional genes were tested using TaqMan Low Density Array (TLDA, Applied Biosystems) using ABI Prism PCR - HT7900 (Applied Biosystems).

see Table 2.

### Primary astrocytes isolation and cell cycle analysis

Primary astrocytes were derived and grown as previously described [54]. For Cell cycle assay, primary astrocytes cells were collected by centrifugation and resuspended in 0.5 ml phosphate buffered saline (PBS) containing 0.1 mg/ml of propidium Iodide (Sigma Cat # P4170) and 0.1% sodium azid. propidium Iodide fluorescence was detected on 5000 cells and determined by fluorescence-activated cell sorting (FACS) analysis (FACSort; BD Biosciences, USA). Quantification of cell cycle phases was analyzed by CellQuest software (BD Biosciences).

## Supporting Information

File S1 **Complete data of whole genome analyses of RNA isolated from the entire brain, excluding the cerebellum, of 3 wild type and 3** ***Eif2b5-*** **mutant mice at postnatal days 1, 18 and 21 using Affymetrix Mouse Genome 1.0 ST microarrays.** RNA from each mouse was analyzed on a separate chip. The differentially expressed genes for each developmental time point (P1, P18 and P21) are presented.
(XLS)

File S2 **Full gene lists of enrichment results of the differentially expressed gene sets.**
(XLS)
